# Baltimore Gynæcological and Obstetrical Society

**Published:** 1888-03

**Authors:** 


### BALTIMORE GYNÆCOLOGICAL AND OBSTETRICAL SOCIETY.

T. A. Ashby, M. D., read a paper on "Syphilis of the Endometrium."

There are few, if any, of the organs or tissues of the body which are not involved in one way or another in the various manifestations of syphilis, when once the virus of this malady becomes a constitutional infection.

That the syphilitic poison has predilections for certain organs and tissues is a well-known fact. The manifestations of this disease are largely influenced by bodily conditions and constitutional states. Thus, anæmia, chlorosis, and general debility favor the outbreak of syphilitic lesions which might have pursued a milder course, or been kept in total abeyance, by a condition of health. Pregnancy is supposed to exercise the same influence upon the syphilitic woman. The character of this influence is modified largely by the age of the syphilis. Thus, a woman who contracts syphilis during pregnancy is affected differently from one who contracts the disease prior to conception. In the first case the predisposition to premature delivery is far less potent than in the second.

Secondary syphilis is almost sure to manifest itself in the syphilitic woman when the disease is contracted prior to conception or during the act of conception. Ohlshausen mentions that among 657 syphilitic women, 231 miscarried, while 426 were delivered at term of living and dead children. Parvin states that at Lourcine 260 aborted out of 416 pregnant syphilitic women.

Abortions induced through syphilitic influence may be brought about either through maternal or fœtal infection. A woman previously inoculated with syphilis will abort more readily than one who contracts the disease during pregnancy, and the danger of abortion seems to be in ratio to the period of infection. Thus, contagion communicated at the time of fecundation is more likely to lead to a separation than where the poison has been introduced after the fourth month. Syphilis may be communicated through the father to the fœtus, which may or may not result in its death and separation, and the mother may escape infection. On the other hand the mothers have become infected through the fœtus. A woman may have secondary syphilis and pass through the entire term of pregnancy without any manifestation of the disease in connection with the reproductive organs, but this condition of exemption may be regarded as an exception to the general rule that syphilitic women are almost sure to abort.

When pregnancy becomes established in the syphilitic subject it invites a manifestation of the disease at the point of contact of the fœtal and maternal tissues. The decidua becomes involved in a condition known as *syphiloma of the decidua*, and the placenta is so modified in its structure and mode of development as to constitute a condition recognized as placental syphilis. The extent of these changes which take place in the decidua and placenta indicates the result of the syphilitic involvement. Separation may or may not occur according to the extent and influence of the poison upon the maternal and fœtal tissues. The placenta may be affected throughout its entire thickness, or only either the maternal or the fœtal portion. When the infection occurs through the mother, the maternal portion is that which is chiefly involved, and *vice versa* when the disease approaches through the fœtus.

The local manifestation of the syphilitic virus in the decidua, as a general rule to which I know of only two exceptions, ceases as soon as separation takes place, whether by miscarriage or labor at full term. The degenerative changes which follow the termination of gestation seem sufficient to remove the involved endometrium. This process must almost invariably take place. I have been unable to find any references in the literature of this subject to a continuation of the syphilitic manifestation upon the endometrium after labor or miscarriage. If observers have noted this condition they have been singularly remiss in calling attention to it. That a condition of continued involvement of the mucous membrance at the placental site does occur, my experience with two cases herein related fully confirms.

The condition observed is one of continued proliferation of epithelial tissue—a highly luxuriant granulation, if I may so term it—which returns again and again after removal, resembling in this respect the proliferative exfoliations of an epithelial cancer. The sole origin of this condition I have referred to secondary manifestations of syphilis in the endometrium at the placental site or in this neighborhood. The disease under consideration has followed, in two cases under my own observation, a miscarriage which was referred to the influence of the syphilitic poison. In case two there was a return of the endometrial trouble, after an interval of three years from date of former treatment, which could have no reference to any other known causative influence. In my opinion syphilis was the entire cause of an inflammatory condition of the endometrium which was followed by hyperplasia of the elements of the decidua, a condition which has been described by De Sinéty as a fibroid degeneration of the villi of maternal syphilitic origin. Why the latent influence of the syphilitic virus should have manifested itself in endometrial involvement I am unable to explain any more than the various other singular anomalies of this disease which do not seem susceptible of rational solution. We can the more readily understand the primary outbreak of secondary syphilis in the decidua and placenta during gestation, and the continuation of the syphilitic influence upon the endometrium after miscarriage, since here we have the possible retention of placental tissue as a probable nidus for the subsequent outgrowth of granulations. In this instance the influence of the poison is simply continued until overcome by local and constitutional treatment.

The subsequent development of syphilitic lesions upon the endometrium I can only account for upon the general assumption of a local dyscrasia in connection with the lining membrane of the uterus, inviting a concentration of the specific influence upon this membrane, which resulted in hypertrophy of the glandular elements and a degenerative change in the epithelial lining of the uterine cavity.

In case one, in which the local influence was continued after the separation of the fœtus, the lesions were localized—that is, in seeming relation with the placental site. In case two, the entire lining membrane of the cavity seemed involved, though the process in this case was more tractable to treatment than in case one.

I present the histories of these cases.

Mrs. A., aged 24 years, primipara, miscarried between the fifth and sixth months of pregnacy. Prior to this event she had been treated by her attending physician for an indurated chancre and subsequent mucous patches on her vulva and labia minora. This disease she had contracted from her husband during the early weeks of married life. She was not informed as to the nature of the affection, and has been kept in ignorance of the specific character of her trouble out of deference to her domestic relations. Following the miscarriage, a portion of the afterbirth was retained, but this was speedily removed with the curette. Hæmorrhage, however, continued for some five or six weeks, and during this time the curette had been employed two or three times, each time removing lumps of degenerated mucous membrane and vegetations. The uterus remained large, subinvoluted, and in a very relaxed condition, and as a result of frequent intra-uterine applications and curetting, a mild metritis was induced, which was followed by elevation of temperature, violent pain, and profuse muco-purulent discharge more or less tinged with blood. Recognizing the specific history of this patient the family physician made use of antisyphilitic treatment with almost negative results. Mrs. A. continued to run down, and by copious losses of blood was greatly reduced in flesh and strength. Her physician losing confidence in his own intelligent treatment of the case requested me to see the patient in consultation, and then insisted upon my taking entire charge of the case. The diagnosis already established was confirmed, and an effort was made to relieve the distressing symptoms, which at this time were referable to the constant and profuse hæmorrhage, subinvolution, uterine colic, and general debility. Ergot, which had previously been administered, was again employed. The uterine cavity was carefully curetted, and large masses of epithelial tissue and vegetating fungosities were removed. Astringent applications, iodoform, tannin, and other agents designed to influence the tissues through local effect, were employed. The result of this method was temporary in its effect. Hæmorrhage would cease for a few days, but the least bodily exercise would cause its reappearance. The granular condition of the endometrium would again and again reappear after constant curetting, employed at intervals of one or two weeks. The tendency to a re-formation or outgrowth of fungous neoplasms was so constant that it was next to impossible to suppress them for a longer time than a few days. In addition to the local treatment, which was heroic enough to answer every temporary purpose, ergot and iodide of potash and general tonics were administered thrice daily in large doses. Mercury had been employed by my predecessor, and I simply gave the iodide of potash. This condition of the endometrium continued off and on for over three months, during which time I employed the curette frequently and made constant applications to the endometrial surface. Finally the tendency to proliferation of fungous tissue began to diminish and I had the pleasure of witnessing a gradual shrinkage in the size of the uterus, a contraction of its walls, and returning healthy condition of the endometrium. The menorrhagia, which continued off and on for over four months, finally ceased and menstruation became normal. It has continued so up to the present time, now five months since recovery. The involution of the uterus is not as yet complete, but the uterine cavity is contracted and more in keeping with the normal shape and size. During the early progress of the case the cavity was so large that it would have contained easily a medium-sized orange.

In this case the tendency to re-formation of granular tissue was more marked than I ever witnessed. In some of its aspects the proliferation of neoplasms resembled a malignant degeneration, but this idea was dismissed and the theory of syphilitic influence was accepted as in full accord with the history of the case.

The explanation seems to be this: Under the influence of specific disease the placenta and decidua were primarily involved and separation took place, which resulted in the miscarriage of pregnancy and the removal of the fœtus and secundines. The decidual membrane remained behind involved in syphilitic disease, and, in its exfoliation, continued to develop neoplastic tissue. The process of degeneration thus established was continued under the influence of the syphilitic virus. As fast as one set of neoplasms was removed a new set came on to take its place, thus continuing the pathological state of the endometrium. I am convinced the influence of iodide of potash was a potent factor in the treatment of this case. This was shown on several occasions. It became necessary several times to discontinue the use of the drug in consequence of its effect upon digestion. During these intervals, whether from a bias in my own mind or actual fact, I was led to believe that hæmorrhage was more severe and the recurrence of the neoplasms was more marked. I had never before witnessed a condition of endometrium at all similar to that present in this case, and I have associated the influence of syphilis with the causation of the condition herein described. In my opinion a non-syphilitic endometrium would not behave in this manner. In cancerous disease a similar condition might be observed, but the recovery of my patient disproves this assumption, whilst her history gives strength to the syphilitic theory. Whilst this case was fresh in my mind a second case came under my care which confirmed the view expressed above in regard to the influence of syphilis upon the endometrium. The extent of the involvement was neither so great nor so intractable as in the case of Mrs. A., but the history of the patient clearly points to a syphilitic influence extending through a series of years and secondarily involving the endometrium after a lapse of some three years.

Mrs. B., aged 27, primipara, was married six years ago. Some six months after marriage she became pregnant and about the sixth month of utero-gestation she miscarried without any assignable cause referable to objective conditions. Subinvolution, menorrhagia and metrorrhagia followed in the wake of the mishap, and for many months the health of this lady was greatly depreciated.

She was treated both locally and constitutionally by several physicians, with the result that hæmorrhage ceased but subinvolution remained. Her husband, a gentleman of considerable intelligence, had contracted syphilis some time prior to marriage, for which he had been treated, with what he presumed to be entire success. Believing himself cured he entered into matrimony only to find the germs of the disease aroused into new activity by the new state. He almost immediately inoculated Mrs. B. with the poison, which became manifest in constitutional disturbance as well as in the local influence upon gestation. Following the miscarriage, which was the first explosive effect of the syphilitic poison upon the part of the latter, both husband and wife were placed under syphilitic treatment by a physician in New York City, to whom they both applied. The wife was kept in ignorance of the nature of her disease, and, of course, that of her husband, and this ignorance now holds. The successive years of struggle with syphilitic manifestations by husband and wife I shall not discuss here, but the history is not an uninstructive one. Outward manifestations were so successfully combated with anti-specific remedies that a complete check was placed on these. With the exception of sore throat, slight loss of hair, and rheumatic pains, the husband has escaped. The wife continued to bear the legacy of an interrupted gestation in the shape of an entailed uterine disease. With the exception of backache, pelvic pain, leucorrhœa, and general debility, no symptoms had occurred during the past three years referable to the uterus until six months ago. She had never conceived during this time. About May last, without any exciting cause, uterine hæmorrhage became profuse at the period, and during the intermenstrual period there was a free loss of blood, lasting over two or three days. Menorrhagia and metrorrhagia were both established. This condition continuing, in spite of the use of ergot and other ecbolic agents, the husband became alarmed, brought his wife to this city, and placed her under my care. Upon examination the uterus was found to be unusually large, flabby, and relaxed. The probe easily entered three and a half inches. The cavity was open and distensible, readily admitting of the free rotation of the sound. The cervix was patulous and eroded, the edges gaping from an old bilateral laceration. The mucous membrane of the cervix and body was highly granular, and bled profusely the moment it was touched. The introduction of the curette brought away large masses of epithelium and gelatinous vegetation, which clearly accounted for the free flow of blood. Hæmorrhage stopped the moment the curetting ceased; in fact before it was complete, for the uterus contracted so firmly under this stimulus that the blood supply was immediately cut off. Within less than a week's time there was a return of hæmorrhage, and the highly vascular condition of the endometrium reappeared. The curette was again introduced and again a mass of epithelial tissue and vegetations was removed. Under this second curetting hæmorrhage again ceased, and notwithstanding the fact that daily applications of tannin and glycerine and iodoform were made to the entire endometrium, the tendency to re-formation of the vegetations went on, and curetting again became necessary. These neoplasms were again removed in less quantity than in the first instance. Local applications were made to the uterine cavity during the three subsequent weeks before the mucous membrane assumed an approximate normal condition.

Iodide of potash was given in 15-grain doses *ter die* during the entire period, and I have reason to believe its influence was direct.

I am unable to account for the condition of the endometrium observed in this case save on the ground of a syphilitic influence. Would a non-specific endometritis behave in this way? I confess my own experience has never presented a case at all similar. Whilst I have frequently met with granular conditions of the endometrium following miscarriages and in the non-gravid state, they have almost invariably responded to treatment when first instituted and have shown no such tendency to continued re-formation of neoplasms as in the two instances cited. The knowledge of a syphilitic history in these cases induces me to refer the influence to the disease in question, and leads me to formulate the law that in all cases of obstinate and persistent endometrial involvement the condition of a syphilitic influence should be questioned.

I cannot but believe that the assumption of a specific influence as an etiological factor in obstinate conditions of endometrial disease may lead to the employment of constitutional treatment *pari passu* with the local which may secure results at variance with our expectations. It is well known that many women have specific disease of which they are wholly ignorant, and we may readily suppose that in a certain number of this class the influence of the specific poison may manifest itself in an impression upon the endometrium either through repeated miscarriages, through catarrhal states inducing, it may be, sterility, or in conditions allied to those described in the cases herein related.

DISCUSSION OF DR. ASHBY'S PAPER.

Dr. WILSON said that he had had similar cases occasionally during his thirty-six years of practice. The facts in one case were in brief as follows: He was called to treat the patient for a persistent metrorrhagia. Under the local use of Churchill's tincture of iodine the patient so far recovered that she was delivered at term of an apparently healthy child. When a few months old this child developed a characteristic syphilitic eruption and died with hydrocephalus. This experience was repeated in the case of a second child. Until some months after the death of her second child the mother presented no signs of syphilis. At that time a general syphilitic eruption appeared, and she never subsequently conceived. Before the first conception mentioned, her physician was unaware of the fact that her husband was a syphilitic.

Dr. BROWNE recalled the case of the wife of a syphilitic man, who, without ever showing any signs of syphilis herself, had several abortions at the third and sixth months. Her uterus was large, flabby, with a tendency to bleed. She was put upon anti-syphilitic treatment, gradually improved, and has since borne healthy children.

Dr. C. O'DONOVAN, Jr., read a paper entitled, "Remarks on the Use of the Manganese Compounds in Menstrual Disorders."

The paper consists mainly in a review of the contributions which have appeared in the various medical periodicals.

Attention was first called to this use of these compounds by Dr. Broadbent, of London, in the Clinical Society Reports, 1869. Next, Drs. Sidney Ringer and William Murrell reported 69 cases in the *Lancet*, January 6, 1883. They reported the drug, permanganate of potash, useful in all forms of intrapelvic atonic relaxation, or engorgement not due to obstruction. They used one-grain pills, giving one pill three times daily, increasing to two pills three times daily.

Dr. F. H. Martin, of Chicago, has published three papers upon the subject, as follows: *The Medical Record*, September 29, 1883; *The New York Medical Journal*, January 24, 1885; and the *Chicago Medical Journal and Examiner*, September, 1885.

In the discussion of Dr. Martin's second paper Drs. Paoli and Havens reported over 50 successful cases.

Dr. E. J. Doering, of Chicago, reports in the *Chicago Medical Journal and Examiner*, April, 1885, 14 cases—8 successes and 6 failures. Dr. O'Donovan, however, thinks that these cases were not well selected.

In the *New York Medical Journal*, July 27, 1886, Dr. Fordyce Barker enthusiastically supports the use of manganese as an emmenagogue. He mentions three well-defined states of women in which the happiest results may be expected:

"1. In young ladies who come to New York to finish their education, leaving a comfortable home for a boarding-school with more or less uncongenial surroundings, and consequent homesickness, with various neurotic ailments, one of which is apt to be suppression of menses.

"2. In women, whether young or old, who have just returned from Europe, in whom the sea-sickness and other discomforts of the ocean voyage have produced suppression. These he cures invariably.

"3. In those women who develope a decided tendency towards obesity when they become thirty, or thereabouts, and who suffer from various disorders, both physical and psychical, as a consequence, in whom catamenia usually disappear, or become very scanty, causing thereby an aggravation of their other troubles. To these, and to his other patients requiring the remedy, it is administered in doses of two grains three times a day, followed immediately by half a tumbler of water."

Dr. C. E. Billington, of New York, in the *Medical Record*, March 6, 1886, reports four cases, appropriately chosen for the remedy, three of which were cured perfectly, and the fourth much benefited after other drugs had failed. All, however, complained of the usual stomach troubles.

In this paper Dr. T. G. Thomas is reported as using the McKesson & Robbins two-grain pill of manganese binoxide, and speaks of the effects of the drug as "the best I have met with."

Dr. A. F. Kerr, of Williamsville, Va., reports in the *St. Louis Courier of Medicine*, 1886, three cases treated successfully, in two of which peculiar symptoms were encountered; one being an epileptic, with amenorrhœa, in whom menstruation appeared promptly, causing a great improvement at the same time in the epilepsy; the other was a negro who had been subject to vicarious menstruation by the nose, and whose epistaxis ceased upon the reappearance of the menses.

Dr. H. J. Boldt, of New York, in the *Medical Record*, May 26, 1886, says that in selected cases, especially after sea-sickness, he prefers permanganate to any other emmenagogue.

Dr. William T. Ellis, of Livermore, Ky., reports in the *American Practitioner and News*, January 8, 1887, 15 cases, all successes. He uses the one-grain permanganate pill of Parke, Davis & Co.

Mr. T. Maury Deas, of Exeter, England, in the *British Medical Journal*, April 18, 1887, mixes the drug into pills with kaoline oinment, and gives it rather more freely than usual, having given three to six grains three times a day for weeks, and relates one instance in which the catamenia reappeared six months after the commencement of the treatment. He concludes that—

1. Permanganate of potash is a useful and safe emmenagogue, and free from the disadvantages which attend some other remedies of the class.

2. Its use may be continued for months without any bad effects, and success need not be despaired of even after many months.

3. Even when it fails as an emmenagogue, it acts beneficially as a general and nervine tonic.

Dr. Bartholow, of Philadelphia, in the *Medical News* (Phila.), November 22, 1884, quotes from two continental authorities its good qualities in amenorrhœa and

"dysmenorrhœa, characterized by scanty menstruation and anæmia." This is in an exhaustive article on "Permanganate of Potash, its Action and Uses," in which he takes the ground that the properties of the salt are due entirely to the nascent oxygen set free in the stomach, and likens it to the effects produced by ozone used therapeutically. From this conclusion, after a thoughtful consideration of the views and experience of the various observers from whom I have quoted, I feel obliged to dissent, especially after remembering that similar results were obtained from other compounds of manganese—the chloride (Broadbent), the binoxide, and the inunction of the oleate—which latter must effectually dispose of the nascent oxygen theory, leaving us no doubt of the fact that the benefit is derived from the manganese alone. Nor need we be at all surprised at this when we remember that in the same elemental group with manganese are included iron and nickel, the multitudinous uses of the former being known to every practitioner, and the latter an almost untried remedy, believed by some to possess powers that must be eventually recognized. I am myself prepared to accept Dr. Martin's idea, that manganese is somehow a powerful stimulant of the nerve supply of the genital system; but whether in its direct effect upon the trophic nerves of those organs, or through the centres in the brain or cord which regulate their functions, I must wait until physiologists may have investigated before reaching any conclusion, using, meanwhile, what knowledge has been derived from clinical experience for the benefit of my patients.

The opinion seems to prevail, in the article cited, that the gastric trouble complained of in using the manganese compounds does not occur if at least one-half cupful of water is taken with them.

In the discussion of this paper, Dr. Ashby reported the case of a healthy, well-nourished young lady, age 23 years, who has suffered all of her menstrual life with amenorrhœa. She menstruates scantily about once in from six to eight months. Three weeks ago he placed this lady on two-grain doses of permanganate of potash, thrice daily. When she had taken her 49th pill menstruation came on. Various other remedies had been employed by other physicians without producing this result. Dr. Ashby called attention to the difficulty of getting the drug in pure form. He had found many of the pills unreliable. By changing from one manufacturer to another he often obtained the remedy in a satisfactory form, as was shown by its influence.

Professor B. B. Browne read a paper on "Electrolysis for Fibroid Tumors and Pelvic Exudations."

He said: For the past twelve years I have used the galvanic and Faradic currents in the treatment of gynæcological cases, many times obtaining the most satisfactory results after the failure of other means.

The greatest objection to the use of electricity in these cases is the difficulty of keeping the batteries, especially the galvanic, in working order.

In 1877 I used the galvanic current with decided success in the treatment of a large sub-peritoneal fibroid tumor which filled the whole pelvis, and extended above the umbilicus. The tumor was nodulated and seemed to be everywhere adherent; the woman had persistent metrorrhagia, for which she had been previously treated with hypodermic injections of ergot as well as its internal use. Galvanism was applied by introducing the positive pole into the cavity of the uterus and placing the negative sponge electrode over the abdomen. The current was made as strong as the patient could bear without producing decided pain. The application was continued for about fifteen minutes at each sitting and was used twice a week for three weeks; the metrorrhagia was arrested after the third application, and the tumor also diminished in size after the first week. The pains and discomfort accompanying it were relieved at the end of the treatment, and the tumor then was less than one-half its original size.

The treatment I pursue at present is more of a radical measure than that above described. I now place the patient under an anæsthetic, introduce the two needle electrodes through the abdominal walls into the substance of the tumor, and gradually turn on the cells until twenty-four are brought into use. I keep on the twenty-four cells for forty-five minutes, then gradually reduce them and turn off the battery and remove the needles. The patient is then put to bed and kept under the influence of opium for 24 hours, or longer if there is any tenderness or pain over the abdomen. She is kept in bed from three to seven days.

If there be menorrhagia I use as the positive pole an intra-uterine electrode introduced into the cavity of the uterus, continuing this for a few short sittings until the tendency to hæmorrhage ceases; then I use the needle electrodes as above described. If we wish to cause the absorption of a fibroid tumor or of an exudation we must place the needles so that the whole of the current shall be localized in the tumor and not diffused by passing through other resisting tissues.

Before using the needles I dip them in a strong solution of carbolic acid. After the needles have been *in situ* for ten or fifteen minutes a yellowish-white foam collects around the negative needle; at the positive pole a whiter fluid sometimes collects. Where the tumor can be readily reached through the vagina it is preferable to insert the needles from this direction.

It is very frequently the case that the positive needle becomes fixed in the tissues, and sometimes it is quite difficult to remove it. We then find it roughened and corroded by the action of the acids which accumulate around the positive pole.

In regard to the kind of galvanic battery that gives the best results in electrolysis, I think the liquid batteries are the best, although the chloride of silver or dry battery is much more convenient to manipulate, and keeps in better condition than the former.

I will, however, relate two cases from many similar ones, showing the effect of electrolysis on fibroid tumors and pelvic exudations.

CASE I. M. H., colored, age 33 years, married ten years, sterile; has not had her menses for three years; abdomen as large as at full term of pregnancy. Suffers great pain in walking and is unable to work; uterine cavity measures four inches; the whole pelvis is filled with a large fibroid tumor that reaches above the umbilicus.

March 5, put her under ether and used the needles through the abdominal walls into the tumor; gradually increased the current to twenty-four cells, and kept them on for thirty minutes; then gradually reduced the current, removed the negative needle, and attached the uterine electrode to the negative pole, inserted it into the cavity of the uterus, and kept it there fifteen minutes, with twenty-four cells in the circuit. The current was then gradually turned off, the patient put to bed, and kept under opium for two days; on the fifth day she got up, and, as there was no pain or tenderness over the abdomen, she was allowed to walk around her room; on the seventh day she returned to her home in the country, with instructions to return at the end of six weeks for another treatment. She returned at the stated time; the tumor had diminished very much in size, the pains in the abdomen were gone, and she was able to walk with comfort, and could do more work than she had been able to do for the past three years. As she had improved so much and as the tumor was continuing to diminish in size, no treatment was made.

About two months after this she returned to the city again, but as she was feeling well and the tumor getting smaller, electrolysis was not used.

CASE II. Mrs. R., age 22 years, married two years, was confined October 5, 1886, instrumental delivery.

Had gathered breast which confined her to bed for four weeks. Seven weeks from date of confinement Dr. W. was called to see her at night and found her suffering with acute pain in the right inguinal region, radiating up to the umbilicus; the pain was very intense, and required constant hypodermics and large doses of opium to give relief. Ten weeks after confinement Dr. W. found upon vaginal examination a large, hard, tender mass posterior and to the right of the uterus which was immovable and fixed by the exudation.

Pain continued for more than four months, during which the usual treatment for such cases was faithfully persevered with.

On April 7 she was put under chloroform, the two needles were inserted into the mass of exudation through the vagina, and twenty-four cells of the battery were gradually turned on and continued for forty-five minutes. The pains throughout the pelvis were somewhat increased for the first two days after the electrolysis, then a profuse discharge continued for two or three weeks; it was free from smell, was not pus, but looked somewhat like the lochial discharge. The pains now left her and she commenced to gain strength. Six weeks after the operation she went north and remained during the summer. Upon her return, in September, Dr. W. made a vaginal examination and found only the slightest remains of the exudation in some thickening of the tissues at its former site; there was not the slightest pain or tenderness in the parts.

DISCUSSION.

Dr. ASHBY gave a very brief description of Apostoli's method of using electrolysis in uterine fibroids, and said that while he is not prepared to say that it is superior to other methods of treating these tumors, he thinks that it is entitled to careful trial in suitable cases.

Dr. B. B. BROWNE said that he had never used electricity in acute inflammatory conditions, but he had never seen its use give rise to inflammation.

He thought the danger from shock necessarily less in his own manner of applying the electrodes, for the entire strength of the current was thus exerted only on the substance of the tumor, and caused its disintegration.

He considered that this treatment for large sub-peritoneal fibroids of the uterus would to a great extent take the place of hysterectomy or removal of the ovaries, the latter being often impossible if the tumor was of large size.

Electrolysis also breaks up adhesions and thus relieves pain, so that, although the tumor may not be entirely removed, the condition of the patient is greatly improved.

